# ‘Elderly years cause a Total dispaire of Conception’: Old Age, Sex and Infertility in Early Modern England

**DOI:** 10.1093/shm/hkv067

**Published:** 2015-07-04

**Authors:** Sarah Toulalan

**Keywords:** old age, sex, reproduction, infertility, sexual dysfunction, impotence, appearance, beauty

## Abstract

Although the history of old age has been studied in much greater detail in recent years, the subject of sexuality in old age remains relatively under-explored. This article examines early modern ideas about old bodies and sex in relation to fertility, to argue that because old bodies were understood as either infertile (post-menopausal women) or sub-fertile (old men) they were therefore characterised as unsuitable, undesirable and inappropriate sexual partners. Perceptions of old bodies, their sexual abilities, desirability and behaviour were remarkably consistent from the sixteenth through to the eighteenth century. The ridiculing of old men and women's sexual behaviour that permeated contemporary culture in stories, ballads and jokes, alongside medical literature that characterised old bodies as sexually unappetising as well as unreproductive, carried the message that sexual activity was not for the old, and in large part because they were infertile.

Fertility was of great concern in early modern England, continuing into the eighteenth century.^1^ This was a time when procreation was the primary—albeit not the only—aim of marriage, promoted by Church teachings and prescriptive literature. A successfully procreative marriage bound couples together in mutual support to nurture their offspring, thereby ensuring social, political, economic and religious stability through securing bloodlines and inheritance. Production of offspring was also a measure of a successful marital union and an important feature of contemporary notions of manhood and womanhood, as men secured their status as potent patriarchs and women fulfilled their social and reproductive destiny through successful childbearing. Men and women were expected to conform to these allotted social, gender and familial roles, and bodies that did not reproduce were therefore disruptive bodies as they resisted conformity to these expectations.^2^ Old bodies in early modern England can be categorised as disruptive bodies in this respect because the old body in early modern thought was particularly associated with barrenness and sterility: post-menopausal old women were understood to be barren while old men were perceived as increasingly less fertile. Ageing was understood to cause deterioration of the sexual and reproductive parts of the body so that the old were thought to be both infertile and physically unfit for sexual activity. Expressions of elderly sexuality were thus overwhelmingly negatively construed and represented at this time.

Historians have identified anxieties about population and the health of the nation as prevalent at this time: Roy Porter and Lesley Hall have described books such as the hugely popular late seventeenth-century *Aristotle's Masterpiece* as ‘pro-natalist’, resonating with contemporary anxieties ‘that the nation was being weakened by underpopulation’.^3^ As this anonymously authored book drew on texts produced in the mid-sixteenth century, pro-natalism was not only a feature of the late seventeenth and eighteenth centuries. Although these later discussions of fertility drew on classical medical literature as well as sixteenth-century Latin and vernacular texts, they nevertheless found a receptive audience as successful reproduction continued to be highly valued; they were congruent with other contemporary expressions of the need for population growth that were linked to economic prosperity.^4^ Although the population was generally growing from the late sixteenth century, it began to fall again in the middle of the seventeenth century and only recovered its earlier level in the second decade of the eighteenth century; life expectancy remained low.^5^ Historians of demography have shown that this temporary decline was due to a combination of continuing high mortality from endemic and epidemic diseases and European conflicts, emigration to the New World, and the late age of marriage which restricted a couple's fertile years.^6^ The growth in population from the early eighteenth century was brought about by an increase in the birthrate caused by couples marrying at an earlier age, so extending their active fertile period, rather than from any improvements in mortality; infant mortality, especially, remained high.^7^ Life—and health—continued to be precarious fuelling persistent anxieties about fertility and perceptions of the need to promote successful reproduction for the general health and prosperity of the nation.^8^ While scholars have consequently investigated issues surrounding fertility to do with sexual practices, abortion, contraception, illegitimacy, pregnancy and childbearing, it is only more recently that they have begun to consider infertility and childlessness, a trend to which this discussion contributes.^9^ This article further demonstrates the centrality of fertility to early modern thinking about bodies and sex and therefore about who were considered to be unsuitable sexual partners.^10^ It also adds to recent scholarship that argues for age as an important category of historical analysis, in this instance specifically in the histories of the body and sexuality.^11^

Although the history of old age has burgeoned in recent years, the subject of sexuality in old age still remains relatively under-explored.^12^ The paucity of sources recording actual experiences of sex in old age in early modern England (and Europe) has led to a focus upon analysis of representations in popular and other literature.^13^ Many explorations of female sexuality in later life pay attention to the stereotype of the ‘merry widow’ who, accustomed to sexual activity through marriage, was unable to exercise self-restraint in curbing her now ‘highly sexed’ nature, and hence posing a challenge to ‘the social and moral order’ of early modern patriarchal society.^14^ Nina Taunton has given this stereotype a positive spin in her discussion of ‘the lecherous old crone’ whose ‘intense’ and ‘insatiable carnal lust’ led to associations with witchcraft and the temptation of sex with the Devil, through which she might regain sexual agency and remedy her loss of sexual power.^15^ Jennifer Panek similarly identifies positive representations of lusty widows on stage, as attracting rather than discouraging suitors, although such desires were clearly nevertheless a subject for comedy.^16^ Joan Hinde Stewart's study of women and ageing in eighteenth-century France notes that the requirement for chastity that was characteristic of women's lives continued into old age, and that only submission to it would guarantee a comfortable and satisfactory old age.^17^ Stewart also points out that French medical texts often associated menopause with an ‘unseasonal sexual urge’ that was harmful to health, potentially leading to uterine ulcers or cancer. This understanding explained older women's lust while at the same time discouraging and condemning it, further underscoring negative attitudes towards female sexual activity in old age.^18^ Older women, both married and unmarried, were also frequently associated with procuration, ‘grooming’ younger women, even their own daughters, for prostitution, having lost their own sexual allure through ageing.^19^ Amy Froide has also pointed to the anxieties about prostitution and social order aroused by never-married women, but argued that menopause may have allowed older women a degree of independence.^20^ Froide seems to imply here that the perception that such women would not now marry and have children also meant that they were no longer regarded as having sexual prospects, within or without marriage, thus side-stepping consideration of older women's sexuality.

Much scholarship about old women's bodies has focused upon the issue of menopause and its physical changes, often to argue that post-menopausal women were likely now to be regarded as old.^21^ Whether or not menopause actually signalled the beginning of old age for early modern women, the termination of a woman's reproductive life that it brought was remarked upon as characteristic of this stage of her life.^22^ Lynn Botelho has argued that this loss of fertility negatively affected women's social status in this life-stage, giving rise to perceptions of old women as disruptive to households when shared with younger kin, no longer serving any useful function, and associated with witchcraft.^23^ Michael Stolberg has drawn attention to the ways in which menopause was a major critical stage in a woman's life, bringing a host of maladies, some, such as menorrhagia, potentially fatal.^24^ More recently, Cathy McClive has shown how medical practitioners found it difficult to establish the boundaries of ‘normality’ in relation to menopause and its associated sterility: it could be a lengthy stage of life ‘incertaine et instable’ with considerable variation between women in the age at which it occurred.^25^ McClive has further noted that the language used at this time, in both medical books and dictionaries, explicitly connected the ending of the monthly purgation with sterility, old age and ill health; women needed to carefully monitor their health to avoid the worst.^26^ Medicine thus constructed old age as a time of life that was subject to a variety of particular physical maladies (or as itself a disease), although careful living might prolong life.^27^ But menopause did not only bring an end to women's reproductive lives precipitating old age. As this article will show, it was also understood as having specific effects upon women's sexual and reproductive parts that would make sexual intercourse physically difficult and unsatisfactory for both women themselves and for their male sexual partners. Understandings of these physiological changes, as well as their loss of generative function, shaped representations of post-menopausal old women as not fit for sex.

Similarly, it has been frequently remarked that the loss of old men's sexual virility generated the ‘well-established and enduring’ stereotype in all sorts of literature of the foolish old man who, lusting after a younger wife but unable to satisfy her desires, was inevitably cuckolded.^28^ As scholars have further observed, male impotence, especially when it led to marital infidelity or childlessness, undermined a husband's social and family position, challenging his manhood and patriarchal authority and posing a threat to social stability more broadly.^29^ Not only did it disrupt marital and familial bonds and, potentially, bloodlines and inheritance, but might also give rise to potentially riotous communal behaviour through shaming rituals such as ‘ridings’ or ‘charivari’.^30^ Taunton argues that May–December couplings are given both comic and tragic emphasis in early modern drama and poetry. Such a marriage was seen as ‘inappropriate’, containing ‘by its very nature … the seeds of dissention between husband and wife’, causing unhappiness, grief, jealousy, humiliation, anger, suspicion, and fear as the satisfaction of sexual desire is sought elsewhere.^31^

Literary scholars have thus paid considerable attention to elderly sexuality in this context, thoroughly examining the trope of the old lover/lecher, or *senex amans*, in early modern drama and literature in which, according to Christopher Martin, sexual desire in old age was ‘reviled’.^32^ Martin identifies a generally ‘malign’ approach and a ‘prevailing impulse to de-eroticize old age’, accompanied by a ‘correspondingly scurrilous indictment of aged sexuality’, as age and beauty are opposed, eclipsing desire.^33^ Similarly Anthony Ellis analysed comic representations of male humiliation in seeking sexual relations to demonstrate how these served ‘to suppress and deny the validity of sexual desire in old age’.^34^ Such representations continued into the eighteenth century when, as Helen Yallop has argued, ‘Sexuality played no part in the construction of aged masculinity’ and there was a deliberate ‘drive to distance the old man from his sexual past’.^35^ In the same vein, Karen Harvey's analysis of eighteenth-century erotica showed how it reflected contemporary ideas about male impotence so that old men received ‘short shrift’ in this genre.^36^ In contrast, however, Kevin P. Laam has emphasised how, while ‘potency’ fails, nevertheless ‘desire lives on despite exterior decay’, and Cynthia Skenazi demonstrated Renaissance male authors' self-awareness of persisting desire and sexual longing in the face of the deteriorating and sexually failing body.^37^ Furthermore, Philip D. Collington also turned the implications of this stereotype around to argue that it was not just that loss of patriarchal authority followed from impotency, but that ‘age-related losses of political power, familial control and social prestige’ led to anxieties about being cuckolded.^38^

This now considerable body of scholarship reveals and reiterates significant early modern anxieties and negative attitudes towards the old and their sexual behaviour that nevertheless merits further and closer investigation. These narratives acknowledged that the old continued to have sexual desires, and ones that they acted upon in seeking out sexual partners, often, but not always, legitimately through marriage. At the same time, however, they strongly conveyed messages that the old should not do so because sex in old age was inappropriate, frequently ridiculous, and even disgusting, as well as posing a potential threat to social order. While anxieties about the consequences for the household, social and political order of male impotence, or of lusty widows desperately seeking sexual satisfaction, have been thoroughly remarked upon, the ideas about old bodies that underpinned them have not yet been fully elucidated. Although Taunton acknowledged that understanding the old woman as ‘lascivious, innately malevolent and harmful to young men’ derived from ‘the physiology of the ancients’, she did not explain how this physiology specifically meant that old women physically could not, and should not, engage in sex.^39^ These assertions about ‘gerontophobic’ attitudes towards sex in old age are rarely accounted for beyond repugnance towards the decaying old body and its loss of beauty, increasing feebleness and impotence, both social and physical.^40^

Helen Yallop does refer to fertility as a factor in negative attitudes towards ‘age-disparate marriages’, and Karen Harvey discusses impotence in the context of eighteenth-century anxieties about fertility and the health of the nation, but neither fully explains what it was in the ageing process that made these bodies impotent and infertile. Harvey does articulate the importance of male seed and fertility but not exactly how seed was implicated in the loss of male virility and potency.^41^ Many scholars reference the prevailing humoral model to explain the physical changes of old age, particularly its drying and freezing effects that produce bodies that are unattractive and unable to perform sexually. However, how these humoral changes specifically affected the sexual and reproductive parts of the old body to produce sexual dysfunction and reproductive failure are never fully explained. This article thus dissects early modern ideas about old bodies and sex, including how ageing had a deleterious effect on the reproductive organs and generative matter bringing sexual dysfunction and infertility, as well as on appearance and hence on sexual desirability. Old men and women thus embodied the signs of their infertility, indicating that they were therefore unfit for sex. These ideas in turn shaped representations of elderly sexuality as inappropriate, disgusting and ridiculous.

As this article focuses specifically on early modern ideas about the sexual body, reproduction and fertility, it draws primarily upon medical writings, both academic and popular. Although the texts cited are from English language editions, they are not limited to those written in English, but include many that originated on the continent in a shared western European medical tradition. Writings on generation consisted of both original works and a large number of translations of European-authored works from both vernacular languages and Latin, the international language of culture and learning. Authors borrowed from each other, circulating ideas and innovations, frequently copied almost verbatim, in a shared culture of medical knowledge.^42^ Although primarily appearing in medical, anatomical and midwifery texts, information and advice about sex, reproduction and childbearing also featured in many other sources that were accessible to a non-medical audience, such as books of advice, ballads, chap-books, almanacs and even jokes.^43^ Knowledge and information about these matters thus circulated at all levels of society, both literate and non-literate, as oral culture and ‘communities of readers’ brought them to those unable to read or without sufficient means to spend scarce resources on even cheap print.^44^ Records of book ownership have shown that men and women without medical training (such as the Sussex merchant Samuel Jeake) owned medical books.^45^ More accessible, simplified, vernacular works also had a wide circulation indicating that the ideas discussed here were not restricted only to those who practised medicine, surgery and midwifery.^46^

Understandings about the composition of the body and how it functioned were based upon the classical model of four humours—blood, yellow and black bile and phlegm. These four humours corresponded to the four qualities of hot, dry, cold and wet, and by analogy to four stages of life—infancy, youth, maturity and old age. The constitution of the body related to the particular balance of humours, which determined sex and other physical characteristics (musculature, quantity of body fat and hair, hair colour). This humoral model remained dominant despite the development of new theories about generation and of new ideas to do with chemical medicine (that disease was caused by malfunctioning chemical processes rather than humoral imbalances or blockages causing corruption or putrefaction).^47^ There was therefore little change in how old bodies were understood in relation to sex and reproduction during this period. Eighteenth-century authors continued to repeat the words and ideas of much earlier sixteenth-century authors (who were themselves repeating earlier classical ideas) even as they modified them to incorporate new discoveries and theories. Ideas about chemical medicine were incorporated into the humoral framework, while from the later seventeenth century references to female seed were gradually replaced by discussions of eggs and ovaries and it was accepted that male seed contained ‘animalcules’ (although the roles of eggs and sperm in reproduction were not fully comprehended until the nineteenth century).^48^

The balance of the humours altered throughout the life cycle: the warm and moist bodies of infancy and childhood became more hot and dry as they grew into maturity, then lost heat and moisture as they grew old, decaying into the cold and dry bodies of old age. Henry Cuffe described the process in the early seventeenth century where, ‘heat without any the least intermission or pause, worketh upon our moisture, and by little and little consumeth it, it selfe also in time decaying’ until ‘declineth our body unto colde and drinesse, till at length death ceaseth upon our bodies’.^49^ André du Laurens' originally French work on old age that was much translated, including into English in 1599, defined life as sustained by the twin pillars of ‘radicalle heate and moisture’, the loss of which as the body aged he directly linked to the decay of seed and hence of its generative quality. Du Laurens told his readers that as death approached the seed became weaker and ‘becommeth so much the more waterish, and in fine changeth altogether into water: even so the radicall heate and moisture waxe weaker and weaker every houre, infected with some adversarie and unlike qualitie’.^50^ Old age was thus explicitly associated with decay and infertility as generative matter itself decayed.

In the historiography of old age there has been much discussion about when old age began and how it was defined.^51^ As Pat Thane succinctly put it: ‘Old age is defined chronologically, functionally or culturally’; but these definitions may vary considerably, with declining capabilities and sociocultural perceptions of old age not necessarily meshing with the measurement of years.^52^ Early modern authors drew on the literature of the ‘ages of life’ to demarcate both its beginning and the further sub-stages that it comprised. The popular, ancient, division of life into seven-year stages meant that chronological old age was consistently calculated as beginning at 50, but as ‘green’ old age when the body might still be strong and vigorous, gradually deteriorating into ‘decrepit’ old age as one gained in years and came nearer to death.^53^ As many scholars have remarked, this organisation tended to universalise ageing from male experiences. Women were likely to experience old age differently, according to the rhythms of their reproductive role, from menarche to menopause.^54^ The physical indicators of old age were brought about by the changes in humoral balance that began around 50; as one author explained, ‘By old people, I Understand such as have attained to fifty yeares age.’^55^ Death came, as we have seen, when both heat and moisture were completely expended. Old bodies, that were increasingly diminishing in ‘naturall’ or ‘radicall heate’, therefore also had insufficient heat for successful generation.^56^ Constitutional differences between individuals meant that the decline into old age, and consequently into reproductive dysfunction, might vary significantly—it was ‘played out in multiple dimensions, and at different rates’.^57^ Du Laurens cautioned that ‘there are some constitutions that grow old very speedily, and others very slowly’, but women grew old ‘alwaies sooner than the male’, as already constitutionally colder. This gender difference was also reflected in their relationship to fertility, which ended before men's, and can be seen through the alterations brought by age to generative matter.^58^

## Generative Matter: Seed and Menstrual Blood

Despite medical historians' assertions that there was a shift away from humoral theory in the eighteenth century, medical books continued to describe and explain infertility—or barrenness and sterility as it was generally termed at this time, for both men and women—within this model. Inability to reproduce was understood to be caused by humoral imbalance when there was no obvious physical cause such as malformed organs of generation. The aged body's changing humoral balance—increasing coldness and dryness—altered appearance and caused barrenness by affecting the reproductive organs and the materials of generation, seed and menstrual blood. The aged constitution brought defects to the reproductive anatomy and its necessary fluids that made old bodies unsuitable for both sex and procreation.

The primary material required for generation was seed, whether contributed only by the man in the Aristotelian one-seed model of generation, or, in the Galenic-Hippocratic two-seed model, by men and women, who both ejaculated seed at orgasm which then mingled to form a conception. Seed was understood to be made from blood through a process of ‘concoction’ that required heat. Old bodies lacked sufficient heat to perfect this process to create seed that was ‘prolifick’ (full of blood) and hence capable of forming a successful conception. In the early seventeenth century, London physician Helkiah Crooke explained that seed was ‘an excrement of the last concoction’ in the process whereby the body extracted nourishment from the contents of the stomach, distributing blood, as aliment/nourishment, into all the other parts of the body. When these parts had taken what they needed, ‘the salter Particles more fit for the Generation of Seed’ were separated from this ‘redde and pure blood’ by heat, mixed ‘into a thin Liquor’, and turned white by ‘the spermaticall vessels and the Testicles’.^59^ For seed to be fertile and fit for generation, sixteenth-century French surgeon Ambroise Paré observed that ‘it must of necessity be copious in quantity but in quality well concocted, moderately thick, clammy, and puffed with abundance of spirits’.^60^

The seed of old men, though, either lacked these essential qualities or was altogether absent. Thus infertility was ‘of the mans part’ when his seed was ‘cold, thinne, waterie and feeble, as is the seede of old and feeble men’.^61^ This was because, ‘by reason of their abated heat’, there was ‘no overplus [of blood] left’ from which seed could be concocted.^62^ As English midwife Jane Sharp noted in *The Midwives Book* (drawing on earlier works by Nicholas Culpeper, Daniel Sennert and Thomas Bartholin), ‘Old age, cold constitutions, diseased, will not make blood’.^63^ This understanding about how seed was produced, and why in old men it was therefore absent or defective, continued to be repeated into the eighteenth century. Joannes Groeneveld, a Dutch physician practising in London, also stated that ‘in old and decrepid Persons, by the Defect of the Natural Heat, and by the Driness of the Spermatick Vessels, by the previous Heat shrunk up and destiture [sic] of a rich Blood, the Seed is altogether deficient’.^64^ Heat was a crucial ingredient in the reproductive process at all stages so its absence caused generative difficulties. ‘Prolifick’ seed, capable of sparking conception, should have had vital heat in three elements: its natural, elemental heat, from the father's soul, and from the sun.^65^ In addition to having insufficient vital heat, the seed of old men was also defective because its thin, watery consistency meant that it could not be retained in the womb to join with female generative matter to form a conception. Paré remarked that the seed of old men had a ‘more liquid and flexible consistence thereof, so that it cannot stay in the womb, but will presently flow out again’.^66^ The seed of old men was thus defective in both quantity and quality.

However, not all old men would inevitably suffer infertility from defective seed, despite the natural decrease in heat that came with increasing age, because it could decline slowly: men of a hotter constitution could remain ‘vigorous in their old Age’ and able to father children into their 70s and 80s, and even later, as authors frequently remarked.^67^ Thus, although the seven-year organisation of the ages of man defined old age as beginning at 50, some authors argued for a longer period of fertility for men, suggesting that it was not until the mid-60s, or the later stages of old age, that procreative heat began to be lost:

force and heat of procreating Children increaseth daily more and more untill 45 yeares, or till 50, and ends at 65. For then, for the most part, the manhood beings to flag, and the seed becomes unfruitfull, the naturall spirits being extinguished, and the humours drying up, out of which by the benefit of heat, the seed is wont to be made.^68^

This passage, and the surrounding paragraphs were repeated almost identically in *Aristotles Masterpiece*, first published in 1684 and reprinted in new editions and adaptations throughout the eighteenth century.^69^ Both books thus cited the example of a Swedish man ‘a hundred years old, who married a Bride of 30 years old’ and ‘had many Children by her’.^70^ While the explanation of the span of the male reproductive years only indicates continuing male virility and potency in early or ‘green’ old age, which lasted until the mid-60s, the example argues for the possibility of its continuation into much later or ‘decrepit’ old age. Humouralism clearly allowed for considerable variation.

Examples such as this one about the extremely aged Swedish man copied from one book to another were not necessarily entirely legendary: examples of late paternity could be found closer to home both in local communities and in stories about the lives of notable individuals, such as ‘Old Parr’.^71^ However, some medical authors suggested that the progeny of older men would be weak and feeble because they were the product of weak seed. Lemnius wrote that ‘Old men being feeble, their spirits small, and their body dry and exhausted of bloody humours’ were unable to perform the sexual act ‘so manfully’, hence ‘that force that comes from them to beget a child is uneffectuall and invalid’. Thus, ‘such as are born in old age, are slender, small, weak, feeble, not tall, and have not so much strength’.^72^ Nina Taunton notes that Robert Burton similarly warned in his *Anatomy of Melancholy* that melancholy old men produced melancholy progeny.^73^ Whether in ‘green’ or ‘decrepit’ old age, old men were understood as increasingly likely to be sexually and reproductively dysfunctional as a consequence of decaying generative matter, even if they did not immediately lose their potency. Such understandings of the reproductive male old body did not encourage positive attitudes towards old men as lovers nor as prospective fathers.^74^

Although many authors referred specifically to men when discussing seed and fertility, others did not specify to which sex their comments applied, or also mentioned the seed of women. In both one-seed and two-seed models of generation, male seed was understood as the principal generative material that acted upon the female matter to form and shape the fetus. Women's seed was characterised in both models therefore as ‘a more imperfect seed’ than men's: as the colder of the sexes, women produced colder, thinner and more watery seed that required men's seed infused with vital heat to engender new life.^75^ Jane Sharp articulated the widely-held belief that both men and women contributed seed to generation, although male seed was superior, when she told her readers that ‘Mans seed is the agent and womans seed the patient, or at least not so active as the mans’.^76^ Hence, in a compilation of works attributed to Thomas Chamberlayne, possibly trading on the association of the Chamberlain family with midwifery, the author could note that a woman might be barren ‘by reason of some fault in the seed, either the woman being too young, or too old’, and François Mauriceau could add, referring to old women, that the seed ‘of the aged is in too small a quantity, and too cold’.^77^ These authors did not specify the age by which a woman's seed was thought to have lost its capacity for fecundation, making no distinction between the earlier stages of old age and the later, perhaps because the cessation of menstruation was recognised as more clearly indicative of the end of a woman's fertility. But where a woman continued to menstruate into her later years, yet did not reproduce, her infertility could be accounted for as resulting from defective seed.

By the eighteenth century, as the theory that women produced eggs from ovaries gained ground, so discussion of production of little or defective seed increasingly referred only to men. Earlier concerns about women's seed do not seem to have been transferred to their production of eggs. However, it may be inferred that age was thought to affect the production of eggs by the ovaries because authors continued to comment on changes to the appearance of the ovaries caused by age. In the early eighteenth century German professor of anatomy and surgery Laurentius Heister and Scottish physician John Maubray repeated much earlier observations about ovaries that ‘in old Women they appear dry; small, and wrinkled, scarcely weighing half a Drachm’, and at mid-century, English physician John Burton concurred that ‘as Old Age advances, they wither and shrivel up’.^78^ French professor of medicine, Jean Astruc, specifically connected the state of the ovaries in old women with their inability to conceive, linking it with the termination of menstruation which was a certain indicator of lack of fertility: ‘in old Women or those who are past forty five or fifty the *Menstrua* are deficient, the *Ovaria* are too rigid, dense and wrinkled; consequently they are incapable of Fecundation’.^79^ At the very end of the eighteenth century medical authors continued to describe the ovaries in old women as withered and to contrast them with those that were still fertile (‘They are a little plump during the time the woman is fruitful’).^80^ The deterioration of the ovaries and their capacity to produce generative material thus began in early old age and continued as women increased in years.

From the late seventeenth century authors set out, discussed and debated the newer theories of egg-producing ovaries in women and animalcules in sperm that had been revealed through investigations by Harvey, de Graaf, Malpighi, Leeuwenhoek and others.^81^ Authors now altered their terminology to reflect these new discoveries, including referring to ovaries rather than to female testes. However, they also continued to quote from the classical authors and to reiterate ideas that had appeared in sixteenth-century texts, using the concepts of heat and cold and of superior male seed to explain reproductive success and failure. The new ideas were thus incorporated into the older frameworks rather than displacing them.^82^ Ideas about the impaired quality and quantity of semen in old men therefore continued to be repeated and were now simply applied to ‘animalcules’ specifically, rather than to seed in general. Astruc, in the mid-eighteenth century described how,

In the *Semen* of all Animals are observed other smaller Animals, which are very active, having a large Head, and sharp and long tail, much like young Frogs. These are very numerous, so that there are thousands of them in the smallest Drop of the *Semen*. In the *Semen* of Infants, younger Animals, or such as are very old, these seminal Animals are few, weak, and languid.^83^

For old women, though, fertility was not governed only by the deterioration in the quantity and quality of her seed, or the state of her ovaries, but by the cessation of menstruation at menopause.

Whether adhering to a Galenic-Hippocratic two-seed model of generation or to the Aristotelian one-seed model, those who wrote about this subject agreed that women's menstrual blood was essential to conception and gestation. It provided matter to the fetus and then subsequently nourished it in the womb during pregnancy. Lack of menstrual blood was thus understood as indicative of a woman's inability to conceive, so once the menses ceased her reproductive years were over. French physician and professor of medicine, Lazarius Riverius listed as the fourth ‘Cause of Barrenness’ for a woman, ‘When the woman doth not yield convenient matter to form the Conception, and to augment the same, depends upon a want of Seed and Menstrual Blood; so over yong women and over old, do not conceive, through want of both those Materials.’^84^ Although the term ‘menopause’ was not coined until the nineteenth century, early modern medical authors discussed this physiological development primarily in the context of causes of female infertility, but also as a cause of illness and disease.^85^ The ‘stoppage of the terms’ was caused by the constitutional changes of ageing: as heat and moisture declined so there was no longer a surplus or plethora of blood requiring regular evacuation through menstruation. Crooke described how ‘the courses cease, because the heate being nowe become more weake is not able to engender any notable portion of laudable bloud, neither yet if there be any such overplus, is able to evacuate or expell the same’.^86^ This understanding about why menstruation ceased did not change significantly into the eighteenth century.^87^ English physician John Freind, in his authoritative treatise on menstruation, *Emmenologia*, first published in Latin in 1703 and translated into English in 1729, placed slightly more emphasis on the drying of the body with age, but nevertheless concurred that there was insufficient blood to be discharged: ‘as *old Age* creeps on, the Humours every Day become both less redundant, and the Fibres of the Vessels grow more rigid and hard; so that a *Plethora* can neither be accumulated at that Age, nor if it be, can it be discharged, because of the *tenacity* of the Vessels’.^88^ Nearly 50 years later William Smellie explicitly connected these changes with those of old age: ‘when the fibres growing rigid, the *Incrementum* is lessened, the evacuation is no longer necessary, nor has the blood force enough to make good its wonted passage into the cavity of the womb. In the same manner are produced the symptoms of old age.’^89^ Menopause was in this way presented as a ‘natural’ part of the ageing process and thus signalled both the termination of fertility and entry into old age.^90^ However, it might not be clear that menstruation had definitively stopped, so that older women could nevertheless believe they were still capable of conception.

Authors generally agreed about the ages at which menopause would occur, ranging from around 44 to 55; many placed it at 50, implicitly connecting it with the onset of old age.^91^ It was invariably noted that it might occur earlier or later than these ages—albeit unusually—as those who were healthy and strong and had not over-indulged themselves might continue menstruating, and hence to bear children, for longer, complicating fixed ages of life categories for women. French *accoucheur* Mauquest de la Motte noted two exceptions, one who ceased early and another who continued after the age of 60:

I have seen a woman whom this evacuation left at the year of thirty-five, without suffering any inconveniency from it, and another who had had thirty-two children at the year of forty-five, at which time her husband died, who had still her *menses* at sixty-one when she died, and was as regular then as she was at twenty-five.^92^

It is likely, however, that these two cases were noted precisely because they were so unusual.

Thomas Denman in the late eighteenth century also noted that menstruation might continue until nearly 60, but concluded that 44 to 48 was ‘the most frequent time of the cessation of the *menses*’ after which ‘women never bear children’.^93^ Other authors cited examples from classical history of exceptional women who had continued to menstruate, and sometimes to bear children, into their 60s and 70s to suggest that environment, diet and constitution influenced such variation.^94^ Menstruation past the mid-50s, though, was usually regarded as not having ‘any natural cause’ and likely to endanger life, occurring ‘by reason of some violent straining, or other violence’.^95^ Cathy McClive has argued that women who claimed pregnancy when past the expected age of menopause were more at risk of accusations of ‘fausse grossesse’ or of ‘substitution d'enfant’ after a pretended birth.^96^ Women for whom the bearing of an heir was hugely important were also likely to suffer from false hope of pregnancy in later life, especially as menstruation became irregular and unpredictable as menopause approached.^97^ Menstruation beyond the usual age of menopause did not mean a continuation of fertility; rather it was likely to be yet another symptom of the ending of the reproductive years as its cause was ‘some affect that is contrary to Nature, which also hinders all conception’ or, potentially, the prelude to deception.^98^ Variation in the age of permanent cessation of menstruation, together with uncertainty about whether a woman had definitively stopped as gaps grew longer between bleedings, potentially indicating illness rather than menopause, meant that a woman's loss of fertility linked to her entry into old age could not be fixed with any chronological certainty.^99^

But it was not simply the absence of menstrual blood as an essential generative material, nor deterioration of the quality of seed or of the ovaries, that determined women's loss of reproductive capability. Also linked to menopause was alteration to the state of the womb which made it unfit for childbearing. The cessation of menstruation not only removed one of the essential generative substances but also meant that the womb now became more dry and cold as the woman's constitution shifted into that characteristic of old age. This increasing desiccation, together with the lack of menstrual blood to moisten it, caused the womb, like the ovaries, to become small and shrivelled. Crooke wrote that ‘in old women because they are dryed and withered, it is but little’.^100^ A dry womb was unsuitable for childbearing and its cause was usually age. Whereas in a younger woman barrenness from a dry womb might be remedied by the prescription of moistening treatments, ‘It is incurable in old Persons’.^101^ English surgeon John Marten therefore continued to express scepticism about any further extension of women's fertility into old age: ‘how a Woman of threescore or more, whose *Courses* have long before left her, her Intellects decay'd and Parts dry'd almost up, should Conceive, is a Mystery’.^102^ Older age was thus characterised for women as an unreproductive time of life because they had passed the full flower of their years in which they had been fruitful and were now withered, dried up and daily losing the vital heat that had made them both ‘ripe’ for conception and sexually vigorous. As Riverius observed, for women, ‘Elderly years cause a Total dispaire of Conception.’^103^

Marriage and sexual intercourse with older women by younger men was thus regarded as unnatural because it had no purpose. In a society and culture in which the primary aim of marriage was procreation, to marry when this was clearly impossible gave rise to discussion by theologians as well as to comment by medical authors. Lemnius particularly condemned young men who married older women for financial gain rather than with the intention of fathering children:

But since women for the most part about the yeare 45, or at the most 50 have their termes stopt, and no hopes are to be had of Children by lying with them, they do contrary to the law of Nature that marry young men, or men that for greedinesse of mony woe and marry such old women. For the labour is lost on both sides, just as if a man should cast good seed into dry hungry lean ground.^104^

This judgement, while asserting the infertility of old women, nevertheless implied that old women could, and did, continue to have sexual relations. However, continuation of sexual activity when a woman had passed her reproductive years gave rise to considerable anxiety and negative representation, even though Puritan theologians such as William Gouge asserted that procreation was ‘not the only end’ of marriage and that marriage in old age was not forbidden, unless there was a disparity of age.^105^ While it is difficult to define the precise number of years that would constitute such a disparity, some ballads indicated—undoubtedly for effect—disparities of over 50 years. In ‘The Young Woman's Complaint’, for example, ‘A Maiden of fifteene … married an Old Man of Seventy-two year’, pairing a young girl in the freshness of her youth with a decrepit old man to her ‘misfortune’.^106^ Gouge did not entirely rule out marriage in old age, because the sexual act also cemented the marital relationship, binding a couple together in continency and for mutual comfort and support. But, like Lemnius, he condemned those who married for the satisfaction of lust with a younger partner as well as the young who married an old person for financial and social gain.^107^ Popular literature, such as the ballad above, conveyed a similar message as the young girl, forced to marry the old man for ‘his wealth’, cautions ‘For sure youth with age will never agree’.^108^

## Sex and the Sexual Organs

Ideas about old men and women and sex were overwhelmingly negative and revolved around perceptions of the old as both sexually unattractive, thus undesirable, and, usually, as sexually incompetent.^109^ Marten in the early eighteenth century declared that marriage in old age was ‘preposterous’, advising that sexual activity was neither healthy nor appropriate as it would leave the old ‘exhausted’. They would also make themselves appear ridiculous—‘forfeiting their gravity and conduct, losing that honour due to their years’—by seeking that for which their bodies were no longer fit, as stories and jokes about cuckolds and old women marrying much younger men attested.^110^ Old bodies were understood as not fully capable of the sexual act because they had lost the necessary vigour and ‘fruitfulnesse’ that was characteristic of the middle, reproductive, stage of life. Sexual desire was also problematic. Although desire might diminish due to the loss of vital heat, it did not necessarily disappear altogether. The desire for sexual activity could continue into old age but was likely to be tempered by the decline in physical vigour and the drying up of the sexual parts, making it more difficult to achieve.^111^ Where sexual ability and desire were perceived to continue into old age, attitudes were often ambivalent and divided according to gender. Whereas some admiration might be expressed towards old men who continued to be sexually active, and especially reproductively successful, attitudes towards sexually active old women were invariably condemnatory, expressing disgust and contempt. While this was partly a result of a perceived loss of physical beauty caused by ageing, it was also because the old female body was more clearly both an infertile body and one that was less able to engage in the sexual act. At a time when sexual activity and pleasure were so closely entwined with reproduction, sexual intercourse that was without possibility of procreation, and potentially unpleasurable, was problematic.

It was often asserted that sex in old age was particularly harmful: it was ‘exceedingly hurtfull and most pernitious’.^112^ The cold and dry constitution of old age was unsuited to the act of sex because ‘it is an bitter ennemye to all drye Natures, so especially it is moste hurtfull to them that besyde drynesse are also cold’.^113^ Heat was an essential part of successful sex, raising desire and producing generative matter capable of sparking conception, but the expenditure of heat in the sexual act consequently depleted the body's vital heat. In the old, whose vital heat was already diminished, and continuing to fade, sexual activity further reduced it and accelerated ageing: ‘Immoderate venerie weakeneth strength, hurts the braine, extinguisheth radicall moysture, and hastneth on old age and death.’^114^ Thus it was advised that ‘Olde men must content themselves with softer Exercises, lest that the small heate which they have, should be spent.’^115^ Men's diminishing vital heat as they grew old meant that they were increasingly likely to suffer from impotence. The poor quality or absence of seed caused by declining vital heat also resulted in reduced desire for sexual intercourse so that it might be avoided altogether, obviating any possibility of reproduction, or brought weak, unsatisfactory, erections preventing successful completion of the sexual act (and perhaps spurring alternative ways of finding sexual pleasure).^116^ The special qualities of seed stimulated sexual pleasure, so semen that was reduced in quantity or defective in quality had a negative effect on desire and virility as well as on fertility.^117^

This association of old men with impotence was a staple of jokes and anxieties about cuckolds, particularly where there was a disparity of age when an older man married a younger woman: she would inevitably commit adultery because he would be unable to satisfy her sexually.^118^ Gouge cautioned against such marriages because ‘they doe many times much faile of their expectation’ causing ‘more griefe and vexation’ than ‘comfort and contentment’.^119^ Authors from the sixteenth century to the eighteenth identified impotence as a particular issue for old men, where, ‘In some the want of Erections are from a fault in the Spirits, as when they are universally weak and languid, as in old Age and Sickness’, while, ‘In others, want of Erections is from a faulty unpreparedness in the *Genital Juice*, falling short of its spirituous stimulating Quality, either from Superannuation or old Age’.^120^ Despite identifying several causes for impotence in old age, authors were keen to reassure their readers that old age did not necessarily mean impotence for all men, and included comments to the contrary, often in the same sentence. Diemerbroeck added, ‘I except some sort of old men, vigorous in their old Age, who at fourscore and fourscore and ten have begot Children’.^121^ Here Diemerbroeck conflated sexual potency with fertility. As Thomas A. Foster has noted, authors of medical and advice literature ‘did not always make a clear distinction between male infertility and impotence’ and were ‘inconsistent in applying these overlapping terms.’^122^ However, some authors did clearly distinguish between them, such as Gouge who asserted that ‘there is great difference betwixt impotencie and barrennesse’.^123^ The difference lay in the fact that sex was still possible for those who were barren, a defect that could not always be detected, whereas impotence was ‘incurable’ and could be discerned ‘by outward sensible signes’.^124^

Despite Gouge's assertion that impotence could not be cured, there were many remedies available to those so afflicted. As impotence was caused by cold, remedies aimed to increase heat and hence to stimulate lust. Ambergris was particularly recommended for the old as it was categorised as ‘hot and dry in the second degree, it warmeth, resolveth, and strengtheneth’.^125^ English apothecary and botanist John Parkinson recommended it because ‘it doth most conveniently agree with aged persons, to warme, comfort, and strengthen their cold decayed spirits, adding vigour and lustinesse to them, and is accounted conducible to venereous actions.’^126^ However, some remedies could be dangerous if taken by the elderly, especially if very strong, such as the aphrodisiac cantharides, or Spanish Fly.^127^ Marten particularly warned ‘Old Letchers’ against taking cantharides ‘thinking to improve their former Prowess’ because ‘the old Man is so soon shatter'd’ and suffers ‘Diseases and Pains, infinitely more Cutting than the Pleasures were before Charming’, or ‘have kill'd themselves … thinking to oblige themselves and their Mistresses, by being stimulated to the Act of *Venery*’.^128^ The bodies of the old were weaker, more feeble, lacking in vigour and hence less suited to sexual activity, a characterisation that continued into the eighteenth century and which was replicated in erotica; Karen Harvey characterises sex with old men as ‘desperate’.^129^ Taking aphrodisiacs to stimulate virility was only likely to exhaust them further, and authors advised that there was no point in old men taking ‘exstimulating Aphrodisiacks’ because it was not possible to stimulate ‘that Seed which is not there’, and furthermore they might rather ‘loosen the Belly’ so soiling both bed and bride.^130^ This author, Swiss physician Théophile Bonet, here also alluded to the laughable nature of such attempted couplings evoked in cuckold literature and jokes about old men and their young brides in describing these remedies as having ‘a ridiculous effect’.^131^ The perception of old men's use of stimulants as ‘ridiculous’ was also mirrored in eighteenth-century representations in erotica of their resorting to flagellation in a desperate attempt, often doomed to failure, to raise their flagging manhood. While, as I have shown, representations of flagellation in seventeenth-century texts were often positive because they enabled erection and ejaculation, so encouraging fertile sex, by the eighteenth century, in relation to old men, such an outcome was clearly understood as less likely.^132^ Despite many authors' assertions that men could remain virile and reproduce well into old age, more usually it was suggested that they would not—and would therefore require medical, or other, assistance. But remedies for the sexual deficiencies of old age might instead be harmful and sexual success was therefore uncertain and potentially pernicious.

Physical unsuitability for sexual intercourse was not restricted only to old, impotent men. In old post-menopausal female bodies the genitals were also unsuitable for sexual intercourse: the vagina, or ‘neck of the womb’, would be too narrow (‘streight’) and dry to allow penetration. Regular menstruation lubricated the sexual parts making penetrative sex easier and less painful, but menopause removed this effect.^133^ Decreasing heat and moisture in old age made the vagina dry and contracted so that penetrative sex was more difficult and painful. Paré described how, ‘In process of age it grows harder, both by use of venery, and also by reason of age, by which the whole body in all parts thereof becomes dry and hard.’^134^ This was also a problem for older women who had not been sexually active previously: Riverius stated that in ‘elderly Virgins … the Genital Parts, … do become withered … and so strait, that they cannot afterwards easily admit a mans Yard’.^135^ Other later authors, both English and continental, repeated these statements almost verbatim. Post-menopausal old women were understood as not only barren but also as having sexual parts that were unsuitable for sexual activity: they were too hard, dry and contracted, lacking the essential moisture and flexibility that was required for successful, pleasurable, penetrative sex.

These physical changes that brought infertility and difficulties in performing the sexual act were also associated with a diminution in sexual desire in women. English apothecary William Drage attributed diseases of the womb caused by ‘A drie Distemper’ to old age and further drew the conclusion that this would diminish sexual desire: ‘in a general driness of the Body, from defect of Humours or old Age, or drying Causes, as Inflamation and Heat, there are sent forth few *Menstrua*, and Seed; the Mouth of the *Uterus* is dry, blackish and clefty, they desire not much Copulation’.^136^ However, old women were not usually represented in popular culture as lacking in desire or disinterested in sex. Some ballads and jokes mocked old women's continued desire for sex and marriage, implying that they should leave this dimension of life to those more suited to it, because it would come to a bad end, often signalled in the ballad title, such as *A merry new Song of a rich Widdowes wooing, That married a young man to her owne undooing*.^137^ As Yallop has remarked, this would be a ‘union of opposing conditions, and therefore doomed’.^138^ Jokes often juxtaposed very old women who married young men in the prime of their early manhood then complained when inevitably left sexually unsatisfied. When reproved for having ‘foolish’ and inappropriate desires, ‘having one Foot in the Grave’, the punchline emphasised the necessity for containing old women's otherwise socially disruptive ungovernable lust: ‘What! … wou'd you have me turn Whore?’^139^ Like prescriptive literature on marriage, and medical warnings of uterine diseases following from continuing sexual activity, such representations served a regulatory function, acknowledging continuing sexual interest in the old while at the same time expressing disapproval.

Despite the trope of the ‘lusty widow’ there seem to have been far more jokes about old men unable to satisfy young wives, suggesting that this was more of an anxiety than old women's continuing lust into old age.^140^ Old men's sexual incapacity had the potential to disrupt marriage, bloodlines and inheritance: an unsatisfied wife might seek sexual satisfaction in an adulterous relationship that could lead to her bearing another man's child. A post-menopausal woman who sought sexual satisfaction, although morally reprehensible and potentially disruptive of family and communal relationships, could not pass another man's child off as her husband's, nor, if unmarried, be a burden on the parish. It is possible that this disparity is also representative of the remarriage prospects of those who had been widowed and the greater prevalence of marriages where the husband was older than his bride.^141^ Older men were more likely than older women to remarry, although Vivien Brodsky has demonstrated that the widows of craftsmen and tradesmen in London had better remarriage prospects as they had financial assets that made them attractive to an ambitious, younger apprentice or journeyman (perhaps giving rise to the strictures of theologians like Gouge against marrying for financial gain).^142^ Jeremy Boulton has subsequently confirmed that ‘in the economy of poorer Londoners, youthful widows possessing even a little property were particularly attractive in the male-dominated marriage market’.^143^ Similarly Amy Froide has identified representations of wealthier singlewomen as still marriageable.^144^ However, the numbers of older women either marrying for the first time or remarrying, especially those who were past, or nearing the end of their childbearing years, were very small.^145^ Some older widows who were able to support themselves were also unwilling to give up their improved legal status or to compromise their children's inheritance, whereas widowers, whose remarriage had no effect on their legal status, were likely to seek a wife to take care of the household and children, and who could support them in their older age.^146^ Whether or not sexual desire continued into older age, making remarriage a desirable prospect, the old were themselves characterised as utterly sexually undesirable.

## Appearance and Sexual Desirability

Old bodies were not only thought unsuitable for sexual activity because of their impaired generative parts and matter, but also because their bodies were altered by the signs of ageing so that they were no longer judged sexually desirable. As well as harmful, sex was regarded as ‘most unseemly and foul in old age’.^147^ This was partly because the qualities needed for successful coitus were lacking in the aged—strength, agility, warmth, moisture—but mostly because the old were perceived to be physically unattractive. Lemnius linked appearance with performance when he asked, ‘Who sees not how uncomely it is for an old man that is full of wrinkles, and worn out, to fall to kissing and embracing like to young people; for old folks are unable to perform those duties.’^148^ External signs of ageing such as baldness in men, increasingly grey or white hair, leanness of body that also brought dry, wrinkled skin, loss of suppleness of movement, and altered body shape were remarked upon negatively.^149^ Medical writers particularly commented on changes to women's breasts as they aged, describing them as ugly, and also useless because no longer fit for suckling infants. One author explicitly linked ugliness to the extinction of sexual desire: ‘Love is nothing else but a desire of Beauty; Ugliness to the contrary is the reverse of that, which at its appearance, becalms our Tempers, checks our Raptures, flattens our Desires, and at once proves to us an Antidote against Lechery.’^150^ Such descriptions were clearly opposed to the characteristics of young, fresh, beautiful and desirable bodies, male and female, so that the idea of sexual activity involving the old was presented as disgusting and unnatural.^151^ One author who wrote on beauty, Thomas Buoni, attributed it ‘to riper yeares’ when its purpose was to ‘stirreth up a desire of generation’, while for those in old age, or ‘decrepite’, beauty was ‘a delightful remembrance of things past’.^152^ Neither beauty nor the sexual activity it incited were to be part of old age. The physical characteristics of old bodies were represented as both sexually repellent and indicative of their infertility so marking them as unfit for sexual activity and procreation.

One of the most immediately noticeable bodily signs of the shift into old age, which also clearly signalled the constitutional shift towards coldness and dryness and hence likely infertility, was alteration to the colour and abundance of hair. Changes to hair brought by age and humoral change were also associated with processes of ‘putrefaction’, a term that evoked both illness and the decay of the body in death. Crooke noted the coarseness of hair in old age, or its loss, caused by increasing dryness: ‘Those that are well in yeares have hard haire, because their skin becomes hard & thicke in old age, by reason of their coldnesse and siccity’. Men grew bald ‘from defect of Aliment’ or ‘for want of hot and clammy moisture’.^153^ Such men were regarded, as Thomas Foster observed, as ‘poor marital partners’ and ‘less than men’.^154^ Hair, particularly the beard, as Will Fisher has pointed out, was ‘a marker of procreative potential’, with hairy men the more virile while those lacking in hair likely to be barren.^155^ Jennifer Jordan has similarly argued that grey hair was ‘the visual indicator of the loss of or decline from manhood’ where manhood was associated with virility and sexual potency.^156^ Change in hair colour to grey and then white was also caused by ‘want of convenient foode’ and because ‘the nourishment wherewith they are fed, is as it were the dregges of Flegme, which in processe of time remaining about the skin, do putrifie’.^157^ Hair became ‘Snow white through Age’ evoking the association of old age with winter and its attendant freezing temperatures, and hence with qualities opposed to successful sex and reproduction.^158^ It also, as Nina Taunton remarks, had ‘effeminising implications’.^159^ Old men and women thus displayed on the most prominent and visible part of their bodies—the head—a clear signifier of both age and a constitutional alteration that was particularly associated with infertility: coldness.^160^ Many books advising self-treatment for common ailments, such as *The Countrymans Physician*, began the section on curing inability to conceive with remedies to rectify barrenness from cold.^161^

For a man, then, baldness, grey, or white hair indicated a potential loss of virility, suggesting that he might be unable to satisfy in the bedchamber, as well as the diminution of sexual attractiveness. As Jordan further suggested, the popularity of the periwig might have had something to do with its ability to mask this external sign of age and its attendant physical decline.^162^ That hair loomed large in the popular mind as signifying youthfulness and vigour is perhaps suggested by its mention in obituaries when an exceptionally old person retained unusually abundant or unaltered hair. In a notice of ‘*An extraordinary instance of Longevity*’ published in the *Norfolk Chronicle and Norwich Gazette* in 1792, it was reported that John Miniken of Cumberland was almost 111 years old but still possessed ‘such an abundance as few people can boast, even in the vigour of youth’. Furthermore, he had, 30 years before, cut off and sold his hair, from which ‘more than 20 wigs’ had been made.^163^ Similarly, it was reported that shortly before 99-year-old Christina Wills died ‘all her grey hair came off and a fine head of fair hair grew … which curled like that of a young person’.^164^ Both abundance of hair and curling hair are noted by Snook as signifiers of beauty at this time.^165^ The retention of hair, of hair colour and curl, were thus regarded as preserving the appearance of youthfulness and hence of warmth and desirability.^166^ Christopher Martin similarly comments on how the maintenance of ‘a youthful self-image’ enables continuing sexuality in old age in poetic representation.^167^ Consequently books of recipes for cosmetics and aids to beauty claimed that their recipes might preserve a youthful appearance. They included recipes for ‘Ointments to restore the Hair’ and to colour it various shades from yellow and red to black.^168^ Swiss physician, Johann Wecker explicitly addressed the desire to remedy the ravages of age and ‘to cover those deformities by which the decay of Time renders a person not only unhandsome, but despicable’. He asked, ‘Why should a woman loose [sic] one of the chiefest Ornaments of her whole frame, a long Train of disheveled Hair, if that can be restored by Art?’^169^ Such restoration brought not only the appearance of youth and beauty, but also sexual desirability with its implication of fruitfulness, although recourse to these measures were frequently mocked and condemned.^170^

Véronique Nahoum-Grappe defines the beautiful female form at this time as ‘curvaceous’ and ‘soft’, thus more rounded and plump than thin. One of the functions of fat was to fill out the body so that it appeared ‘plump, equall, soft, white and beautifull’.^171^ Increasing desiccation made old bodies more lean as the flesh was reduced. London herbalist and translator of many Latin medical works Nicholas Culpeper described this alteration in his translation and commentary on *Galen's Art of Physick*: ‘The drier the Temperature is, the slenderer is the man, and the more Flesh recedes from its due temper to driness’.^172^ Although this loss of flesh applied equally to men and women, medical authors commented particularly on its effect on women's appearance and attractiveness. Bartholin's *Anatomy* concluded that ‘decrepit old Women are deformed, for want of Fat.’^173^ The female old body that lost its soft, rounded form as dry cold humours became dominant, diminishing flesh, was therefore characterised as lacking beauty and sexual desirability.^174^ This perception continued into the eighteenth century as Stewart has confirmed in her study of women in eighteenth-century France.^175^ Furthermore, great leanness of body was also strongly associated with infertility.^176^ Old bodies were further made ugly through alteration to body shape as cold dry humours took hold: the body lost the strength and suppleness of youth becoming ‘hard and stiffe’, mis-shapen, ‘crooked, … heavy and unweildy’, and increasingly deformed.^177^ As humoral balance was associated with both beauty and health, imbalance was therefore regarded as indicative of ill health that could alter the appearance, reducing or erasing beauty; ugliness might then further be associated with moral depravity.^178^ Although David Turner similarly discusses the association in the eighteenth century of very thin bodies with ill health, lack of beauty and moral defect, he does not mention its close connection with both old age and infertility.^179^

The lack of flesh that was characteristic of old bodies also diminished beauty as loss of flesh brought dry, wrinkled skin: ‘Some Authors will have the Fat contribute not only to the Nourishment of all the Parts, … but also unto Beauty, for Persons who have little or no Fat have their Skin dry and subject to wrinkles.’^180^ As David Turner has observed, ‘Advice literature on personal appearance emphasised the importance of an unblemished complexion’ with wrinkles regarded as not only ‘signs of mortality’ but also as ‘the embodiment of declining powers’.^181^ Books of cosmetics and aids to beauty thus also included recipes for potions and waters to remove such deformities and to ‘*make the face youthful*’. These aimed to remove wrinkles by application to the face or through washing, such as the recipe that advised, ‘Take of the Decoction of Bryony and Figs, each alike quantity, & wash the face with it.’^182^ Such advice was aimed at both men and women, indicated by references to ‘he’ and ‘him’ in one such recipe:

Take of Live-sulphur one ounce, Olibanum, Myrrhe, each two ounces, Ambergreece six drams, powder and mix them adding a pint of rose-water, then distil them and keep the water in a vessel well stopt; which if any one will use, at night when he goes to bed let him wash his face, and in the morning with spring water, and it will be most comely.^183^

As we have seen previously, one ingredient in this recipe—ambergris—had the properties of warming and strengthening, and was particularly recommended for the old as an aphrodisiac. Such recipes demonstrate the crossover between cosmetic recipes and medicine in what Snook has called ‘beautifying physic’, that was ‘part of the medical marketplace and the unlicensed practice of early modern healing’.^184^ Although Naomi Baker has argued that women were more likely to be characterised as physically revolting than men, the recommendation of such recipes for men and women suggests that both were conscious of the ravages brought by ageing and desired to counteract them, although Turner asserts that in this increasingly lucrative market, ‘most products were aimed at women’.^185^

One particular aspect of the ageing body and its sexual and reproductive function associated only with old women, and which was presented by medical and other authors as especially unattractive, was changes to the breasts.^186^ Alteration to their appearance was caused by reduced body fat. Whereas the breasts of young women who were ‘ripe’ for reproduction were described as firm, round and smooth, those of old women had the opposite appearance. Heister's *Anatomy* described beautiful breasts as having ‘a moderate Bigness, a due Distance, a tender and white Skin, a Substance somewhat hard, not flabby or pendulous, and a rosy Nipple’.^187^ Pierre Dionis' observation that ‘When Women approach to Fifty years of age, their Breasts wither; and the older they grow, the softer and flaggier are their Breasts, till at last nothing is left of 'em but the Skin’ is representative of descriptions from the early sixteenth century to the late eighteenth.^188^ Numerous authors explained that the breasts lost their ‘smoothnesse & equality’ in old age, ‘the Fat and Glandules being all consumed’, so that ‘onely the skin and the nipples do remaine’.^189^ They also repeated throughout this period the same descriptions of changes to women's nipples as they aged. Diemerbroeck wrote of the nipple or ‘Teat’ that ‘*The colour of it is red in Virgins, more livid in those that give suck; but in Women that are past Child-bearing it grows black*’.^190^ Crooke, earlier in the century, noted that the skin of the nipple was ‘more rugous and unequall’, while Bartholin commented that ‘Only in old women it grows thick.’^191^

Some authors, such as Bartholin, linked form to function, connecting the role of the breasts to nourish the infant with its rounded shape:

The *Use* thereof is, to turn Blood into Milk. And the use of the fat of the Dug is to encrease heat, and to make the Dug of an even round shape. And therefore such as have the Fat consumed by some Disease or old Age, they hang ill favoredly like empty Bladders, and are unfit to make Milk.^192^

When no longer able to conceive, an old woman's breasts also altered and became unfit to nourish a new life. Just as remedies were prescribed for baldness, greying hair and wrinkles, so authors also offered them for sagging breasts to restore the appearance of youth, beauty and fruitfulness. Wecker listed recipes for ‘*A Liniment to smooth flaggy, wrinckled Breast’* and several for ‘*An Ointment for Breasts that hang down*’. Most of these recipes included ingredients that were moisturising and binding to fill out the flesh (frankincense), that might clear the skin to make the breasts more firm (bean-flower), that could help infirmities of the breast (raisins) or of the lungs (dry figs, tragacanth).^193^ The flaccid, appearance of the ageing breasts, with their darker, thicker nipples, were not only judged ugly, reducing a woman's sexual desirability, but were yet further visual indicators that her reproductive organs were equally dry and shrivelled so that sexual intercourse might be more difficult, potentially unpleasurable, and almost certainly non-procreative.

## Conclusion

Perceptions of old bodies, their sexual abilities, desirability and behaviour were thus remarkably consistent throughout these three centuries and were thoroughly informed by the understanding that they were inevitably infertile bodies. Medical writing that associated old age with sexual dysfunction and infertility underpinned and reinforced the regulatory and disciplinary strategies of other contemporary literature. It was an important component of a pro-natalist culture in which bodies were categorised and defined according to their reproductive possibility, which promoted remedies for infertility where appropriate, and discouraged sexual activity in those deemed unfit. Social, economic and political stability could only be ensured through a fertile and reproductively successful population; bodies that were sexually dysfunctional and infertile were disruptive not only of contemporary gender norms, but also of stability more broadly. This was most particularly the case when old, infertile men and women married a younger partner still capable of reproducing, thus depriving a younger woman of her maternal destiny or a younger man of his role as a potent patriarch.

Shaped by humoral physiology that categorised old bodies as ever more cold and dry as they approached death, old men and women were characterised by qualities that were aligned with decay, putrefaction and death and that were explicitly opposed to growth, fertility and life. Male and female generative matter diminished in both quality and quantity, and, in the case of women's menstrual blood, usually ceased to flow altogether; if it did continue to flow it was a sign of disease rather than of continuing fertility. Old women's generative organs dried up as their reproductive function terminated making penetration more difficult, while the impaired quality of old men's seed diminished virility, often causing impotence. Old bodies with their bowed, mis-shapen and emaciated frames lacking in strength and flexibility, their coarse, thinning, grey or white hair and dry wrinkled skin embodied sexual disability, unattractiveness and undesirability. Old female bodies further displayed their infertility and diminished sexual allure in their flattened, sagging breasts that were no longer capable of nourishing an infant. The aged thus embodied infertility, from decaying generative matter and genitals that were not fully functional to body shape, size and appearance. As such, they self-advertised their unsuitability as sexual or marital partners, engendering mockery and ridicule when displaying continued sexual interest that was likely to disappoint and frustrate a younger partner causing familial and social disruption.

## Funding

This research was generously supported by the British Academy [SG-41058] and by the Welcome Trust, grant number WT085433.
